# Blood-based DNA methylation marker model for short-term and long-term lung cancer risk prediction

**DOI:** 10.1186/s12916-026-04973-y

**Published:** 2026-06-06

**Authors:** Megha Bhardwaj, Yi-Qian Sun, Clara Frick, Ben Schöttker, Oluf Dimitri Røe, Bernd Holleczek, Xiao-Mei Mai, Hermann Brenner

**Affiliations:** 1 Clinical Epidemiology of Early Cancer Detection, German Cancer Research Center (DKFZ), Heidelberg, Germany; 2 German Cancer Consortium (DKTK), German Cancer Research Center (DKFZ), Heidelberg, Germany; 3 Department of Clinical and Molecular Medicine, Norwegian University of Science and Technology, Trondheim, Norway; 4 Department of Pathology, Clinic of Laboratory Medicine, St Olav’s University Hospital, Trondheim, Norway; 5 Center for Oral Health Services and Research Mid-Norway (TkMidt), Trondheim, Norway; 6 Heidelberg Medical Faculty, Heidelberg University, Heidelberg, Germany; 7 Cancer Clinic, Sykehuset Levanger, Levanger, Norway; 8 Saarland Cancer Registry, Saarbrücken, Germany; 9 Department of Public Health and Nursing, Norwegian University of Science and Technology, Trondheim, Norway; 10 Cancer Prevention Graduate School, German Cancer Research Center (DKFZ), Heidelberg, Germany

**Keywords:** lung neoplasms, DNA methylation, epidemiology, screening

## Abstract

**Background:**

Screening heavy smokers with low-dose computed tomography (LDCT) has been shown to reduce lung cancer (LC) mortality, however, identifying the specific high-risk population that benefits most, a critical requirement for implementing effective and cost-efficient screening, remains challenging.

**Methods:**

We developed and validated a blood-based DNA methylation marker model (BBDMM) for all participants, including both ever and never smokers, using the LC risk-informative CpG sites from epigenome-wide association studies (EWAS). The model was developed and internally validated in 2,459 participants from ESTHER, a population-based cohort from Germany. Subsequently, BBDMM was externally validated in exactly same 233 participants drawn from the Norwegian HUNT2 and HUNT3 cohorts with long- and short-term follow-ups and cases identified up to 18 and 6.7 years before LC diagnosis, respectively.

**Results:**

The BBDMM predicted LC incidence with an area under the curve (AUC) of 0.84 [95% confidence interval (95% CI), 0.80-0.87] in the derivation set. In the independent external validation sets, AUCs of 0.85 (95% CI, 0.80-0.90) and 0.85 (95% CI, 0.80-0.90) were observed in HUNT2 and HUNT3, respectively.

**Conclusions:**

The BBDMM identified future lung cancer cases with promising potential and the model discrimination was highly stable at different time points. These markers may contribute to the evolution of a blood-based test for predicting LC risk.

**Graphical abstract:**

**Figure:**
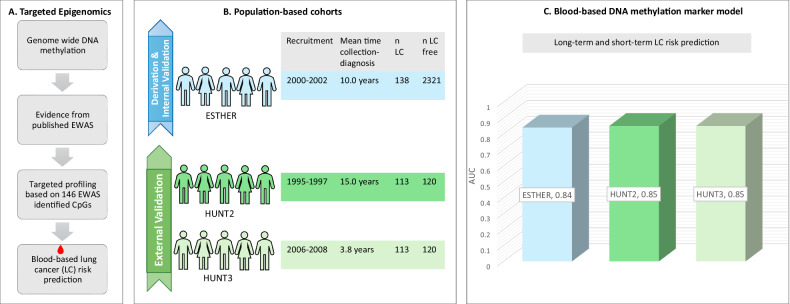
Using prediagnostic blood samples from participants of large population-based cohorts from Germany and Norway, we identified, evaluated and validated a blood-based DNA methylation marker model with equally good prediction for long-term and short-term lung cancer risk.

## Background

Lung cancer (LC) is the most common cancer and leading cause of cancer mortality globally, accounting for more than 2.4 million cancer cases and over 1.8 million deaths in 2022 [1]. Screening heavy smokers by low-dose computed tomography (LDCT) has been shown to reduce LC specific mortality [2–4**]**. A recent Cochrane meta-analysis of randomized trials reported that screening with LDCT was associated with 21% reduction in LC mortality and 5% reduction in all-cause mortality [5]. Multiple LC risk prediction models have demonstrated effectiveness at identifying individuals for whom LDCT screening could be particularly beneficial [6–10]. Traditionally the LC screening by LDCT and these risk models have been mostly restricted to current and former smokers, due to their elevated risk. Lung cancer is associated with various risk factors, nevertheless, cigarette smoking remains the most prevalent, reported in 75% to 95% identified cases [11, 12]. Many countries are yet to implement LC screening policies even for high risk groups, partly due to ongoing debates about the benefits and potential harms of LC screening for defining the targeted population [13]. However, the proportion of never smokers among LC cases is increasing in many countries and up to 25% of LC cases are reported in life-long never smokers [14, 15]. Currently, never smokers are excluded from eligibility criteria for screening under the mainstream guidelines in USA and Europe [16, 17].

Major efforts are made to provide new opportunities for personalized risk stratification and screening prioritization with inclusion of molecular biomarkers [18**–**21]. While traditional screening guidelines have targeted ever-smokers, researchers are exploring biomarkers and other risk factors to help guide screening incorporating never-smokers. Recent epigenome-wide association studies (EWAS) have reported sparse sets of smoking dependent and independent DNA methylation at Cytosine-phosphate-Guanine (CpG) sites associated with lung cancer risk [22**–**30]. In the current study, we aimed to assess up to what extent a model based on the previously reported EWAS based LC risk associated CpGs, may predict future LC risk amongst both ever and never smokers in middle and older age individuals. For deriving and independently externally validating the findings, the participants were selected from population-based cohorts from Germany and Norway, using data and samples from the ESTHER, HUNT2 and HUNT3 cohorts, respectively. In this study, we assess the prediction potential of long-term and short-term LC risk. Predicting long-term LC risk is crucial for early identification of individuals, allowing timely interventions such as lifestyle changes or targeted surveillance.

## Methods

### Study design and study population

The model based on LC risk-informative CpG sites was developed in a two-step approach, with selection of CpGs and construction of a multimarker algorithm in a derivation set that included participants from the population-based ESTHER study. The independent evaluation and validation of findings was performed in external validation sets comprising of exactly same participants from HUNT2 (long-term follow-up, methylation markers up to 18 years before LC diagnosis) and HUNT3 (short-term follow-up, methylation markers up to 6.7 years before LC diagnosis).

#### Derivation set

**Fig. 1:**
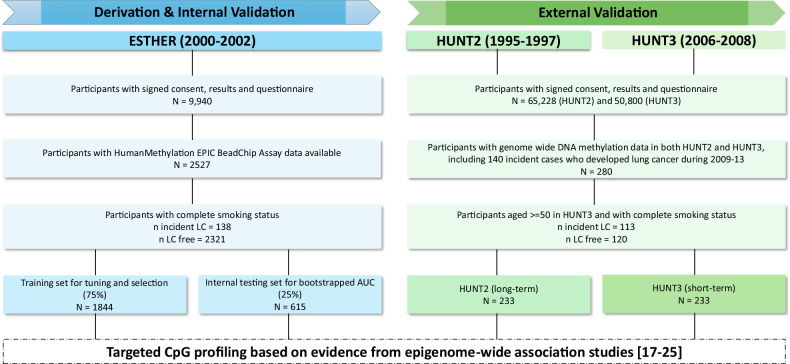
Selection of study participants from ESTHER, HUNT2 and HUNT3 population based cohorts. Abbreviations: LC- lung cancer

The derivation set consisted of participants from the ESTHER study (German full name: *Epidemiologische Studie zu Chancen der Verhütung, Früherkennung und optimierten Therapie chronischer Erkrankungen in der älteren Bevölkerung*), which is an ongoing cohort study conducted in the entire federal German state Saarland, located in south-west Germany. Details of the ESTHER study have been reported previously [31]. Briefly, in the context of a general health screening examination, 9,940 men and women aged 50-75 years were recruited between 2000-2002 and were regularly followed-up thereafter. At baseline information on sociodemographic characteristics, lifestyle factors, smoking behavior and health status was obtained by standardized self-administered questionnaires. Additionally, medical diagnoses and current drug use were obtained from medical records of the general practitioners. Biological samples (blood and urine) were collected and stored at −80 °C until analysis. The prevalence and incidence of all types of cancer cases at baseline and follow-ups was determined by record linkage with data from the Saarland Cancer Registry (https://krebsregister.saarland.de/). The ESTHER study was approved by the ethics committees of Heidelberg University (Heidelberg, Baden-Wuerttemberg, Germany) and of the state medical board of Saarland (Saarbrücken, Saarland Germany). All participants provided written informed consent. The derivation and evaluation of the model in the current study was based on 138 participants who were diagnosed with LC and 2,321 randomly selected participants without LC diagnosis during 21 years of follow-up (Fig. 1). Among the incident cases, the mean time from blood collection to LC diagnosis was 10.0 years.

#### Validation sets

The validation sets included participants from the HUNT Study (Trøndelag Health Study), which is one of the largest population-based health surveys conducted in Norway. Details of the HUNT study have been published previously [32]. Briefly, all participants aged 20 years or older in the northern area of Trøndelag in four waves (HUNT1, 1984-1986; HUNT2, 1995-1997; HUNT3, 2006-2008; HUNT4, 2017-2019) were invited and information was obtained through detailed questionnaires (including information on smoking behavior), health interviews and clinical measurements. In the context of a previous nested case-control study, genome-wide DNA methylation was analyzed in pre-diagnostic blood samples that were collected within HUNT2 and HUNT3 for the identical participants [26]. For the current study, only those who participated in both HUNT2 (long-term) and HUNT3 (short-term), and had complete smoking-related data available were selected. Incident LC cases were ascertained based on the linkage of data between HUNT and the Cancer Registry of Norway. Blood samples were collected at baseline from both the cases and the controls and stored at −80 °C for later use at the HUNT-biobank [33]. The validation of the model was eventually based on 113 incident cases who developed LC during 2009-2013 and 120 age- (+/− 3 years) and sex-matched controls. Among the incident cases, the mean time from blood collection to LC diagnosis was 15.0 years in HUNT2 (range: 11.8-18.0) and 3.8 years in HUNT3 (range: 1.0-6.7).

### Preselection of CpGs from published EWAS and meta-analysis

A systematic search was performed across PubMed database to identify EWAS associated exclusively with lung cancer, and the studies reporting CpGs associated with only smoking and not lung cancer as outcome or studies published in non-English languages were excluded. As shown in Supplementary Table 1, a total of 146 unique sites were identified from the nine published EWAS or meta-analysis of EWAS [22**–**30].

Methylation of DNA extracted from whole blood samples collected at recruitment were quantified using the Infinium HumanMethylation EPIC BeadChip 850K Assay (Illumina Inc., San Diego, CA, USA) according to manufacturer’s instructions in ESTHER, HUNT2 and HUNT3. Probe intensities were normalized using a quantile normalization method. Preprocessing was performed by excluding the probes with detection p-value > 0.01, probes with missing values > 10%, probes targeting the sex chromosomes, cross-reactive probes and polymorphic CpGs. For the assays performed across different processing batches, batch-effect correction using ComBat was employed. Of these 146 CpGs, after normalization and data pre-processing, the methylation beta values of 123 CpGs were available for further analysis.

### Statistical analyses

In the derivation set, comprising of ESTHER participants, the association between each CpG site and LC risk was evaluated using univariate logistic regression models. To account for multiple testing, the effective number of test method was employed [34]. For further analysis, the CpGs were considered only if their p-value was less than 0.05 divided by the number of principal components. For developing a multi-marker model, Elastic Net (EN) tuning was performed where the parameter alpha was optimized via ten-fold cross-validation. This step aimed to determine a stable global regularization strength rather than perform feature selection. For feature stability selection, 1,000 different datasets were randomly generated, in which only 75% of participants were included as the training set. Final risk-informative CpGs were defined as those that were selected in at least 80% of the 1,000 training sets and EN with an optimally tuned alpha and lambda was applied to ensure application of both L1 (Lasso) and L2 (Ridge) penalties. Subsequently, a risk model was developed in each training set and the remaining 25% case-control participants were used to generate overoptimism-corrected estimates by averaging the area under the receiver operating characteristic curve (AUC). The full derivation set was used to build the final model in the form of a blood-based DNA methylation marker model (BBDMM) for predicting future LC cases and generate the apparent AUC estimates. The derived model was subsequently evaluated externally in the independent external validation sets consisting of HUNT2 and HUNT3 participants. The measured methylation at two distinct time points, long-term (HUNT2) and short-term (HUNT3) before LC diagnosis, allowed for assessment of the discrimination potential stability of BBDMM over time. Additionally, the AUC (95% CI) and Odds ratios (95% CI) per 1 standard deviation increases were evaluated to assess the LC risk prediction potential of BBDMM among population subgroups defined by age, sex, body mass index (BMI) and smoking status. Since smoking is the strongest predictor of lung cancer, it was assessed by how much the BBDMM enhances risk prediction beyond merely knowing an individual’s smoking status. This was done by comparing the AUCs of models including BBDMM alone, smoking status alone, or the combination of BBDMM and smoking status as predictors.

All statistical analyses were performed with statistical software R language and environment (version 3.5.3, R core team) [35] using R packages ‘ComBat’, ‘ComplexUpset’, ‘dplyr’, ‘glmnet’, ‘ggh4x’, ‘ggplot2’, ‘minfi’, ‘poolr’, ‘pROC’ and ‘tidyr’. Statistical testing was two-sided, and p-values < 0.05 were considered to be statistically significant.

## Results

The selection of study participants from all the three cohorts is illustrated in Fig. 1. The characteristics of the participants at the time of recruitment in the study’s derivation and validation sets are presented in Table 1. The derivation set consisted of 138 incident LC cases and 2,321 participants without LC diagnosis from the ESTHER study. The validation sets included 113 participants with LC diagnosis and 120 controls free of LC from HUNT2 and HUNT3 with a long- and short-term follow-ups, respectively. The participants were older at baseline in the derivation set comprising of ESTHER (median age: 62.0 and 63.0 years) and validation set comprising of HUNT3 (median age: 67.3 and 67.0 years), as compared to the validation set comprising of HUNT2 (median age: 55.8 and 55.6 years). Among LC cases, 84.8%, 94.7% and 95.6% were ever smokers, and 52.9%, 77.9% and 58.4% were current smokers at the time of blood sample collection in ESTHER, HUNT2 and HUNT3, respectively. Males represented 66.7% of LC cases in the derivation set and 58.4% of LC cases in both validation sets.

**Table 1:** Characteristics of the participants at the time of recruitment

Characteristics	ESTHER (N = 2459) Derivation		HUNT2 (N = 233) Validation		HUNT3 (N = 233) Validation	
	Incident LC cases N (%)	Random sample free of LC N (%)	Incident LC cases N (%)	Random sample free of LC N (%)	Incident LC cases N (%)	Random sample free of LC N (%)
	138	2,321	113	120	113	120
**Age (years)**						
<60	47 (34.1)	727 (31.3)	74 (65.5)	84 (70.0)	16 (14.2)	26 (21.7)
≥60	91 (65.9)	1,594 (68.7)	39 (34.5)	36 (30.0)	97 (85.8)	94 (78.3)
Range (years)	50.0-75.0	50.0-75.0	40.7-68.0	38.8-68.4	52.5-79.7	50.0-79.5
Mean (SD)	61.8 (5.9)	62.8 (6.7)	56.0 (7.0)	55.2 (7.3)	67.2 (7.0)	66.4 (7.3)
Median	62.0	63.0	55.8	55.6	67.3	67.0
**Gender**						
Female	46 (33.3)	1,316 (56.8)	47 (41.6)	51 (42.5)	47 (41.6)	51 (42.5)
Male	92 (66.7)	1,005 (43.3)	66 (58.4)	69 (57.5)	66 (58.4)	69 (57.5)
**Smoking status**						
Never smoker	21 (15.2)	1,210 (52.1)	6 (5.3)	55 (45.8)	5 (4.4)	51 (42.5)
Former smoker	44 (31.9)	729 (31.4)	19 (16.8)	35 (29.2)	42 (37.2)	48 (40.0)
Current smoker	73 (52.9)	382 (16.5)	88 (77.9)	30 (25.0)	66 (58.4)	21 (17.5)
**BMI**						
<25 kg/m^2^	40 (29.0)	510 (22.0)	60 (53.1)	50 (41.7)	47 (41.6)	39 (32.5)
≥25 kg/m^2^	98 (71.0)	1,811 (78.0)	53 (46.9)	70 (58.3)	66 (58.4)	81 (67.5)

LC- lung cancer; N- number; SD- standard deviation

**Fig. 2:**
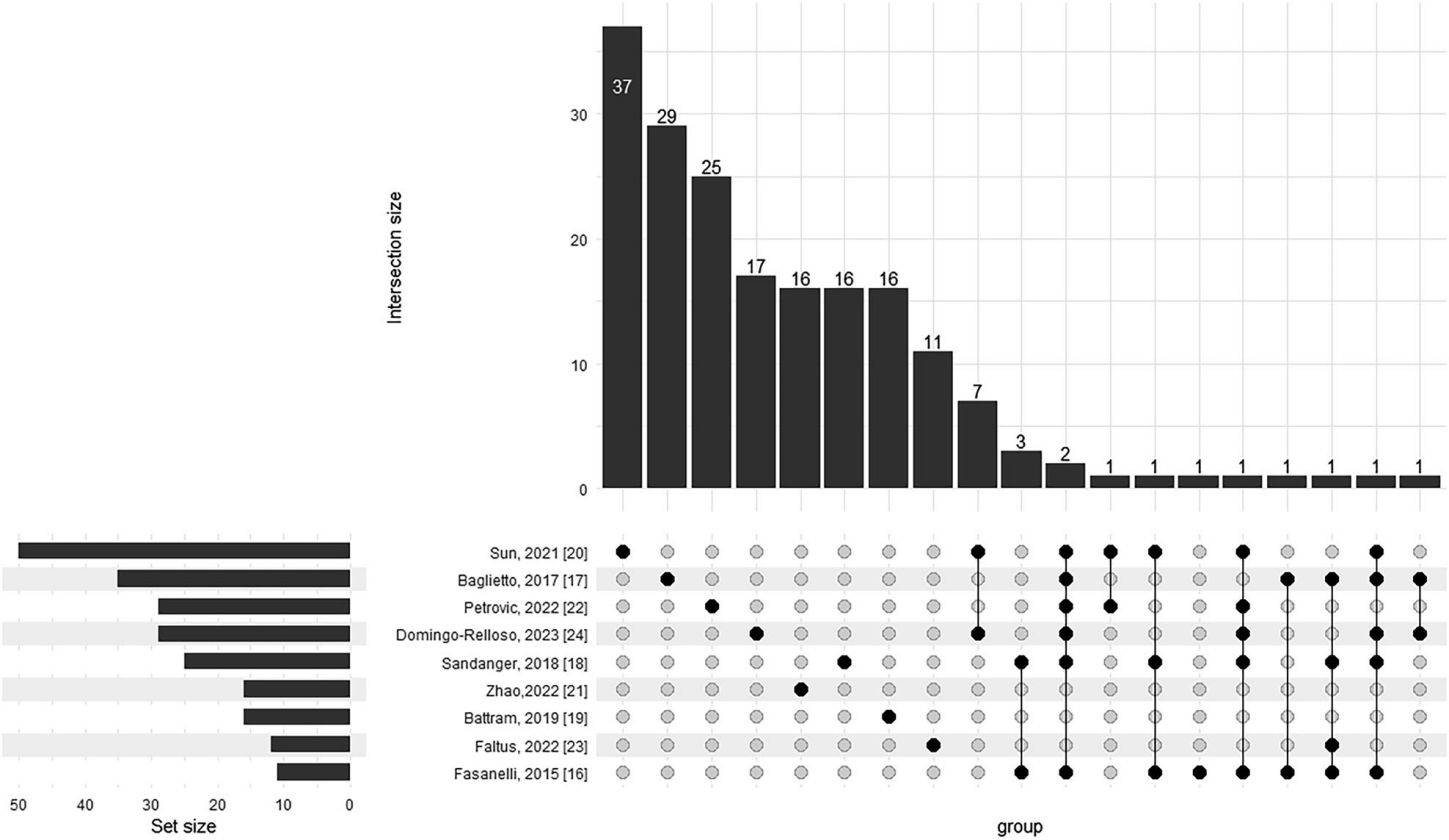
The intersection of biomarkers across different epigenome-wide association studies [22**–**30]. Set size reflects the total number of biomarkers in each individual study (left). The rows correspond to studies and the columns represent specific intersections (right). Filled dots in a column indicate which studies are part of that intersection and these are connected by lines to highlight multi-study combinations

**Fig. 3:**
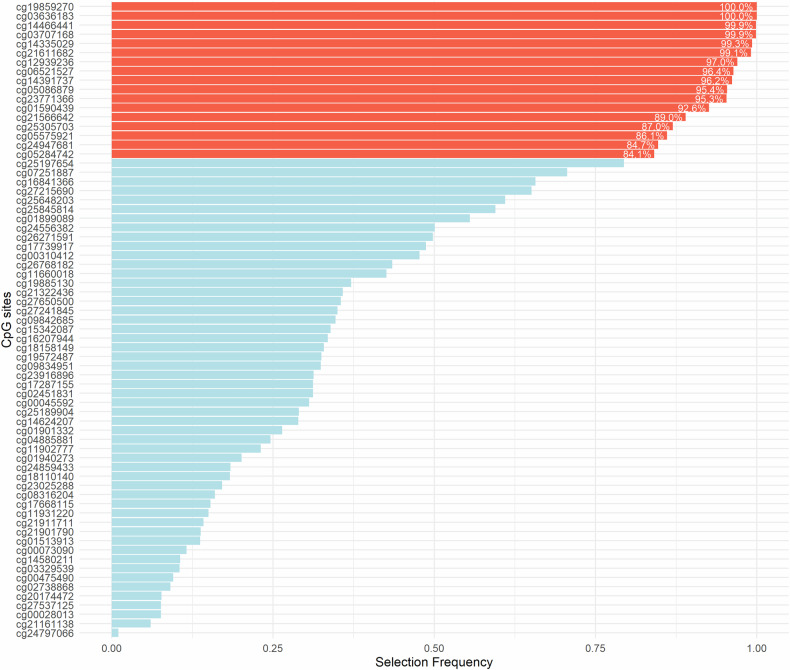
Feature selection stability depicted by the proportion of times the CpGs are selected in 1000 training datasets by ELASTIC NET regularization for developing the blood-based DNA methylation marker model (BBDMM). CpGs with selection frequency of ≥ 80% are marked in red

Of all the unique CpGs identified from published EWAS for LC risk prediction, only some overlapped across different studies (Fig. 2). The hierarchical cluster heatmap depicting methylation values of all unique CpGs across ESTHER participants is presented in Supplementary Figure 1. As shown in Fig. 3, 17 CpGs that were selected in at least 80% of 1,000 re-samplings were included in the BBDMM (blood-based DNA methylation marker model). The 17 selected CpG sites annotating 13 genes as presented in Table 2 were cg03636183 (*F2RL3*), cg05575921 (*AHRR*), cg21566642, cg14391737 (*PRSS23*), cg19859270 (*GPR15*), cg14466441, cg21611682 (*LRP5*), cg23771366 (*PRSS23*), cg03707168 (*PPP1R15A*), cg05284742 (*ITPK1*), cg12939236 (*BMF*), cg14335029, cg05086879 (*MGAT3*), cg25305703 (*CASC21*), cg24947681 (*THBS1*), cg01590439 (*LINC00051*) and cg06521527 (*NEDD9*). For the selected 17 CpGs, the violin-scatter-box plots (Supplementary Figure 2) display full distribution and central tendency, that reveal site-specific methylation differences among incident LC cases and participants who did not develop LC from ESTHER, HUNT2 and HUNT3.

**Table 2:** Individual CpGs selected in the blood-based DNA methylation marker model that was derived in the ESTHER study (listed by ascending p-values)

CpG sites	Chromosome	Gene	Smoking dependent	Coefficient (95% CI) from the final model	p-value*
Intercept	**-**	**-**	**-**	−3.608 (−3.909, −3.335)	<0.001
cg03707168	19	*PPP1R15A*	yes	−0.247 (−0.680, −0.172)	<0.001
cg19859270	3	*GPR15*	yes	−0.236 (−0.506, −0.098)	<0.001
cg21611682	11	*LRP5*	yes	−0.192 (−0.697, −0.118)	0.01
cg14466441	6	*Intergenic*	yes	−0.167 (−0.506, −0.057)	0.01
cg05086879	22	*MGAT3*	yes	0.093 (0.049, 0.512)	0.02
cg05284742	14	*ITPK1*	yes	0.072 (0.028, 0.653)	0.03
cg06521527	6	*NEDD9*	yes	0.102 (0.028, 0.437)	0.03
cg14335029	9	*Intergenic*	no	−0.177 (−0.440, −0.009)	0.04
cg03636183	19	*F2RL3*	yes	−0.266 (−0.683, 0.005)	0.05
cg05575921	5	*AHRR*	yes	−0.118 (−0.577, 0.271)	0.05
cg12939236	15	*BMF*	yes	−0.092 (−0.332, 0.026)	0.09
cg24947681	15	*THBS1*	yes	0.106 (−0.041, 0.511)	0.09
cg23771366	11	*PRSS23*	yes	−0.105 (−0.475, 0.050)	0.12
cg25305703	8	*CASC21*	no	0.089 (−0.053, 0.434)	0.13
cg01590439	8	*LINC00051*	no	−0.083 (−0.331, 0.055)	0.16
cg14391737	11	*PRSS23*	yes	−0.174 (−0.475, 0.171)	0.37
cg21566642	2	*Intergenic*	yes	−0.137 (−0.465, 0.191)	0.41

^*^p-value associated with the coefficient estimate for each CpG from the final model

**Fig. 4:**
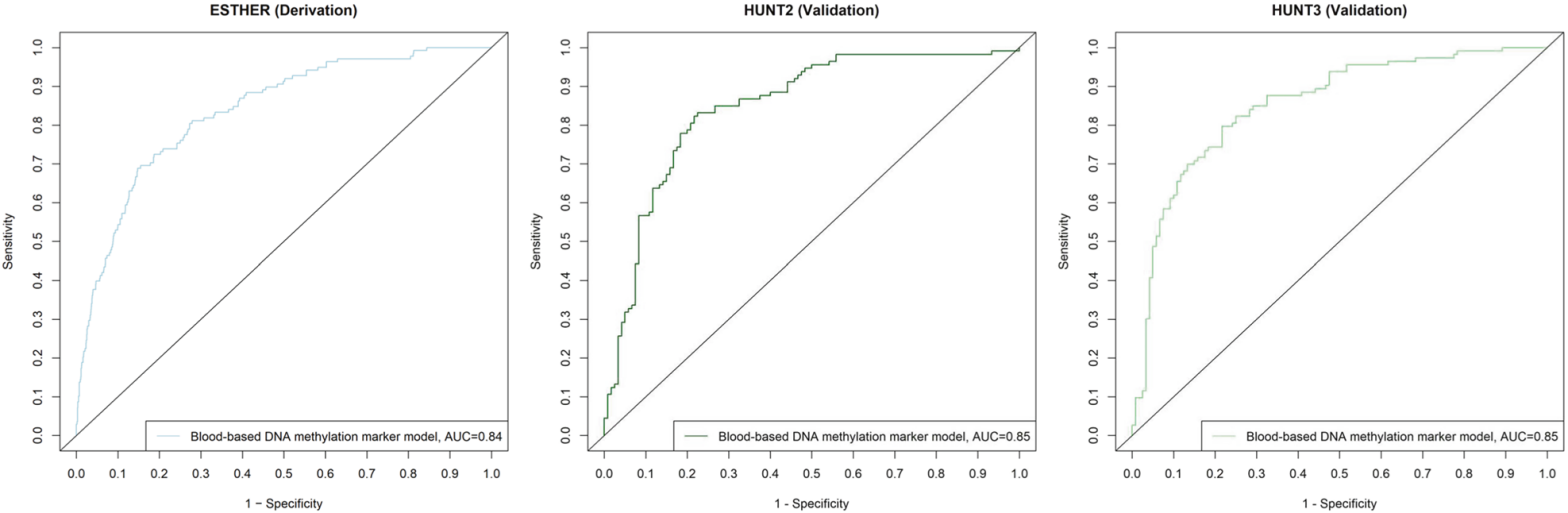
Area under the receiver operating characteristic curve of the blood-based DNA methylation marker model (BBDMM) overall in the derivation and validation sets

The performance of the BBDMM comprising of the 17 CpGs for predicting LC cases are presented in Table 3 and Fig. 4. Overall, the BBDMM predicted the LC cases in ESTHER with an apparent AUC of 0.84 (95% CI, 0.80-0.87) and the overoptimism corrected AUC of 0.82. For external validation, the performance was evaluated in the independent validation sets and AUCs of 0.85 (95% CI, 0.80-0.90) were observed in participants from both HUNT2 and HUNT3, respectively. Overall, the discrimination of the BBDMM was stable across time in long-term and short-term samples, with equivalent AUC of 0.85, supporting the robustness of the methylation markers over time.

**Table 3:** Ability of the BBDMM to predict lung cancer incident cases

Group	ESTHER (Derivation)			HUNT2 (Validation)			HUNT3 (Validation)		
	n total	n incident LC	AUC (95% CI)	n total	n incident LC	AUC (95% CI)	n total	n incident LC	AUC (95% CI)
**Overall**	2,459	138	0.84 (0.80-0.87) 0.82*	233	113	0.85 (0.80-0.90)	233	113	0.85 (0.80-0.90)
**Age**									
<60 years	774	47	0.86 (0.81-0.92)	158	74	0.86 (0.80-0.91)	42	16	0.82 (0.69-0.95)
≥60 years	1,685	91	0.82 (0.78-0.87)	75	39	0.83 (0.73-0.93)	191	97	0.86 (0.81-0.92)
**Sex**									
Females	1,362	46	0.83 (0.78-0.88)	98	47	0.81 (0.71-0.90)	98	47	0.81 (0.72-0.90)
Males	1,097	92	0.82 (0.78-0.87)	135	66	0.88 (0.82-0.94)	135	66	0.88 (0.83-0.94)
**BMI**									
<25.0 kg/m^2^	547	40	0.85 (0.80-0.91)	110	60	0.86 (0.78-0.93)	86	47	0.84 (0.75-0.92)
≥25.0 kg/m^2^	1,909	98	0.83 (0.79-0.87)	123	53	0.83 (0.75-0.90)	147	66	0.86 (0.80-0.92)
**Smoking status**									
Never	1,231	21	0.80 (0.71-0.87)	61	6	0.61 (0.26-0.95)	56	5	0.70 (0.48-0.92)
Ever	1,111	117	0.79 (0.75-0.84)	172	107	0.78 (0.70-0.85)	177	108	0.80 (0.73-0.86)
Former	773	44	0.78 (0.71-0.86)	54	19	0.66 (0.51-0.82)	90	42	0.79 (0.70-0.88)
Current	455	73	0.74 (0.67-0.80)	118	88	0.69 (0.57-0.81)	87	66	0.71 (0.56-0.86)

AUC- area under the curve; BBDMM**-** blood-based DNA methylation marker model; LC- lung cancer; n- number; 95% CI- 95% confidence interval

*- Bootstrap adjusted estimate of area under the ROC curve

In the ESTHER and the HUNT participants, the predictive performance was similar across age related subgroups (Supplementary Figure 3). Within the overweight and obese subgroup (BMI ≥ 25 kg/m^2^), the AUCs were estimated at 0.83, 0.83 and 0.86 in ESTHER, HUNT2 and HUNT3 participants, respectively. Amongst the male participants, the BBDMM predicted future LC cases with AUCs of 0.82, 0.88 and 0.88 in the participants from the three studies. Comparable predictive performances were seen for the never-and ever smokers in the derivation set, with AUCs of 0.80 and 0.79, respectively. AUCs for never smokers in the validation sets were lower, but these have to be interpreted with caution as they were based on just 6 and 5 LC cases, respectively. As presented in Table 4, overall, the ORs per increase in the BBDMM by 1 standard deviation for LC risk were 3.47 (95% CI, 2.90-4.16), 4.88 (95% CI, 3.30–7.21) and 5.30 (95% CI, 3.50–8.01) in the derivation and both the validation sets, respectively. For the participants age 60 years and older, the ORs were 3.13, 4.43 and 5.82 in the derivation and two external validation sets, respectively. Amongst the female participants, ORs of 2.87, 3.58 and 3.84 were observed in the ESTHER, HUNT2 and HUNT3 participants, respectively. For the subgroup with BMI < 25 kg/m^2^, the ORs were 3.97, 5.39 and 4.87 in the three cohorts. The BBDMM improved the predictive ability of smoking status by 0.100, 0.050 and 0.090 in the ESTHER, HUNT2 and HUNT3 participants, respectively (Supplementary Table 2).

**Table 4:** Odds ratios per 1 SD for lung cancer in relation to BBDMM for predicting lung cancer incidence in the derivation and validation sets

Group	ESTHER (Derivation)	HUNT2 (Validation)	HUNT3 (Validation)
	OR (95% CI)		
**Overall**	3.47 (2.90–4.16)	4.88 (3.30–7.21)	5.30 (3.50–8.01)
**Age**			
<60 years	4.64 (3.14–6.86)	5.32 (3.24–8.74)	4.07 (1.70–9.72)
≥60 years	3.13 (2.55–3.83)	4.43 (2.25–8.74)	5.82 (3.61–9.41)
**Sex**			
Females	2.87 (2.24–3.69)	3.58 (2.10–6.07)	3.84 (2.23–6.61)
Males	3.58 (2.79–4.61)	6.65 (3.70–11.95)	7.53 (3.94–14.39)
**BMI**			
<25 kg/m^2^	3.97 (2.71–5.80)	5.39 (2.97–9.75)	4.87 (2.55–9.31)
≥25.0 kg/m^2^	3.31 (2.69–4.06)	4.29 (2.56–7.20)	5.49 (3.21–9.39)
**Smoking status**			
Never	2.17 (1.61–2.92)	1.27 (0.58–2.80)	2.00 (0.75–5.29)
Ever	3.40 (2.67–4.34)	3.13 (2.10–4.65)	3.49 (2.30–5.29)
Former	2.97 (2.16–4.10)	2.06 (1.09–3.87)	3.78 (2.04–6.99)
Current	2.67 (1.91–3.74)	2.17 (1.38–3.42)	2.11 (1.26–3.55)

BBDMM**-** blood-based DNA methylation model**; **OR**-** odds ratios; SD**-** standard deviation; 95% CI- 95% confidence interval

## Discussion

In participants of large population based prospective cohorts of screening age adults, we assessed if and by how much methylation of LC associated CpGs in whole blood DNA could select people at risk of LC. Using blood samples collected years before diagnosis, we developed and externally validated a DNA methylation marker based risk prediction tool. A BBDMM comprising of 17 CpGs predicted future LC cases with an AUC of 0.84 in population-based German ESTHER cohort. The prediction of the BBDMM was persistently stable over time in samples collected 11 years apart, with AUCs of 0.85 each in the Norwegian HUNT2 and HUNT3 cohorts. Our findings suggest that this model may well potentially serve as complementary risk prediction tool to the existing modalities and contribute to efficient selection of LC risk individuals in the middle-aged and older general population for screening, thereby enhancing the screening efficacy.

Even though never smokers are excluded from screening eligibility criteria, an increasing number of cases are identified in never smokers [14] and following the LC mortality reduction from annual LDCT screening for those at high risk in the randomized trials, research is underway to assess whether never smokers could similarly benefit. However, there is concern regarding use of routine screening for a broader low-risk population without strong proof of benefit [36] and there have been no randomized controlled trials performed to fully assess the benefit and harms of screening people who have never smoked. Preliminary findings of a study on an Asian population indicate that never smokers with certain risk factors (e.g., family history or environmental exposures) may have LDCT detection rates similar to the smokers [37]. Measuring DNA methylation markers could be valuable, even in never smokers, because epigenetic alterations have been shown to associate with future LC risk [38]. In the current study, we establish a BBDMM based on LC risk informative CpGs for never, former and current smokers combined, that demonstrates robust performance in predicting future LC cases. The identified model for predicting future LC cases showed promising potential even amongst never smokers in ESTHER. Even though the risk discrimination potential among never smokers seemed lower in HUNT studies, this result needs to be interpreted with caution given the limited number of nonsmoking LC cases (n = 6) in HUNT2 and (n = 5) in HUNT3 and also the resulting very broad confidence interval of the AUC estimate. The performance of BBDMM in never smokers remains yet to be fully established, largely due to the limited sample size available for this subgroup, which constrains definitive conclusions about its predictive accuracy. Nevertheless, the current findings provide a useful initial indication of its potential. Further validation in larger population-based cohorts with a greater number of never smokers will be important to confirm the model’s reliability for risk prediction in this population. Such studies would enable a more precise assessment of the generalizability of the BBDMM in never smokers, a subgroup for which current research is still evolving.

In the context of LC screening, blood-based biomarkers are becoming increasingly integral, offering a minimally invasive and efficient supplement to traditional tools. The blood samples used in the current study were drawn years before LC diagnosis (up to 21 and 18 years in ESTHER and HUNT2, respectively), inferring the observed methylation differences could be reflective of the modulations, immunological changes or epigenetic programming occurring early on in LC development [39]. The inclusion of samples long before diagnosis helps guard against reverse causation and ruling out influence of preclinical disease on the methylation markers. The methylation measurements were also evaluated at two points, in samples with a shorter follow-up of up to 6.7 years (HUNT3) and longer follow-up of up to 18 years (HUNT2), strengthening the temporal design. Replication of associations in samples 11 years apart demonstrates the reliability, stability credibility and robustness of the methylation markers. However, there was no increase in predictability in the shorter time to LC diagnosis, probably reflecting that the epigenetic changes are early and stable occurrences in the carcinogenic process. Predicting long-term risk of LC can enable timely preventive measures, support personalized screening for high-risk individuals, reduce unnecessary procedures for low-risk populations and inform public health planning by projecting future disease burden.

As DNA methylation is frequently one of the earliest alterations in carcinogenesis process, these markers are emerging as promising contenders in the context of screening and early detection. In this study, we assessed the predictive potential of LC risk associated smoking dependent and independent CpGs. Some of the most promising biomarkers like the cg03636183 in *F2RL3*, cg05575921 in *AHRR*, cg21566642, cg14391737 in *PRSS23* and cg14466441 in intergenic region were included in BBDMM that was derived, internally and externally validated in the current study. CpG sites such as cg21566642, cg14391737, cg14466441 and cg19859270 have been identified as potential predictors for LC risk, however, their clinical utility remains preliminary. Hypomethylation at cg05575921 (*AHRR*) and cg03636183 (*F2RL3*) are robust biomarkers for LC risk that add information beyond smoking history [20, 40, 41]. Though methylation at cg05575921 and cg03636183 is not independently causal in the absence of smoking, single point mediation analysis from a recent study reported that *AHRR* and *F2RL3* genes explain ~37% of the total effect of smoking in lung cancer [30].

In the current study, we identified and validated a model comprising of several smoking dependent and independent CpG sites that could predict future LC cases with high accuracy. A potential shortcoming of the measurement of the specific methylation markers identified in the current study for selecting high- and low-risk smokers is the requirement of blood sampling and laboratory analyses which go along with additional efforts and costs, whereas “traditional” heavy smoking classification and LC risk models can be applied based on self-reports only. Although DNA methylation measurements in our study were based on technically demanding and costly genome-wide methylation arrays, the measurement of DNA methylation at a smaller number of 17 or less CpG sites could be implemented by much simpler, low-cost technologies in mass screening. Although traditional heavy smoking classifications are easier to apply, they are potentially subjective to recall and social desirability bias, as among the participants of the current study, whereas a biomarker-based tool may provide an objective and reliable assessment. The most effective way to enhance selection of those at LC risk could be to employ direct measurements of the best predicting DNA methylation markers, possibly along with other promising blood-based biomarkers (proteins, microRNA and ctDNA) and risk factors, and we encourage to further evaluate their implementation in a clinical trial environment among high-risk and low-risk groups under real life conditions.

This study employed a multi-stage design and meticulously advanced statistical approach to develop and internally validate a DNA methylation marker model in the participants from ESTHER and then externally validate findings in the HUNT2 and HUNT3 participants. The CpG sites included in the current study were identified from EWAS that published blood-based DNA methylation markers associated with LC. The DNA methylation based prediction model was developed and internally validated in the ESTHER cohort, i.e., in a sample that was completely independent of all the cohorts included in the originally published EWAS. The risk prediction potential of some of the identified markers like *AHRR* (cg05575921) methylation and *F2RL3* (cg03636183) methylation in whole blood DNA has previously been demonstrated in independent cohorts including a lung cancer screening trial cohort [40**–**42]. The results from the current study reflect that the DNA methylation markers could enable improved selection of individuals for LC screening. Future research should aim for further optimization and validation of approaches to identify right high-risk as well as low-risk people for LDCT screening. To our knowledge this is the first study that utilized CpGs strongly associated with future risk of LC from EWAS using the Illumina HumanMethylation450 BeadChip array, as well as the Illumina HumanMethylation EPIC BeadChip 850K array with a wider coverage. Given that methylation was assayed in same individuals from HUNT2 (long-term) and HUNT3 (short-term), the identical discrimination of BBDMM, supports the stability and reproducibility of the markers.

Even though the current study provides evidence of the potential of using LC risk associated CpGs for risk assessment, it should be noted that while the list of 146 candidate CpGs used to derive the model drew upon published EWAS from multiple cohorts like, NSHDS, NOWAC, MCCS, EPIC, CLUE and others, 34 CpGs of these were exclusively from HUNT. In the present study, the entire model development comprising of regression analysis, selection stability and final model weight estimation was conducted solely in ESTHER, an independent cohort which was not used in any previously published EWAS. Only after development of model in ESTHER, the fixed model was applied to the HUNT2 and HUNT3 for validation without any further tuning. Nevertheless, the prediction performance observed in HUNT2 and HUNT3 may be considered as slightly optimistic and future research would benefit from validation of this methylation marker model using prediagnostic samples in independent larger populations with detailed subgroup analysis. Our analysis exclusively focused on risk stratification for selecting people for LC screening, a key design feature of any LC screening program. While further research is needed to validate the clinical utility of biomarkers within screening trials, such trials are restricted to participants that were preselected by previously employed selection criteria. Therefore, both components, analyses of risk stratification within population cohorts in the screening eligible age range, and analyses of clinical usefulness within randomized clinical trials encompassing high- and low-risk populations are needed for comprehensive validation. The final proof of superiority of biomarker supported selection of LC screening participants with respect to LC mortality reduction might come from randomized trials comparing selection with and without consideration of the biomarker of interest. However, such results will not be available in the near future, and informed decision on how to select participants for LC screening programs currently in planning in many countries should be based on the best evidence available to date, to which these results may make an important and timely contribution. Though the current study did not evaluate optimal screening time and intervals, these findings provide a strong foundation for the integration of biomarker-based risk with time-to-event models. The clinical utility could be improved through the incorporation of such information, allowing precise risk stratification and personalized, risk-adapted screening, with timely interventions such as LDCT provided for high-risk individuals while unnecessary procedures are minimized for those at lower risk.

## Conclusions

This study developed and externally validated a model that has potential utility for selection of individuals at risk of LC for targeted screening. The BBDMM performed equally well for short-term as well as long-term LC risk prediction and these markers could enable improved selection of individuals for LC screening. Future research studies should evaluate the potential for further enhancement of risk prediction, as well as the feasibility, effectiveness and cost-effectiveness of implementation of enhanced risk stratification in LC screening.

## Supplementary information

Supplementary Figure 1: The cluster heatmap depicting β-methylation values from low (blue), intermediate (white) to high (red) in ESTHER participants by age, sex and outcome subgroups.

Supplementary Figure 2: Scatter-box-Violin plots showing distribution of the DNA methylation markers selected in the models among incident lung cancer cases and participants who did not develop LC from A. ESTHER, B. HUNT2 and C. HUNT3 population based cohorts.

Supplementary Figure 3: Area under the receiver operating characteristic curve of the blood-based DNA methylation marker model (BBDMM) among population subgroups by A. Age, B. Sex, C. BMI and D. Smoking status in the derivation and validation sets.

Supplementary Table 1: EWAS identifying CpGs associated with LC risk

Supplementary Table 2: Ability to predict incident lung cancer cases

## Data Availability

Due to restrictions of informed consent, the ESTHER study data cannot be made publicly available. However, use of the data from the ESTHER study for collaboration projects has been and will remain the approach for data sharing. Data from the HUNT Study that are used in research projects will, when reasonably requested by others, be made available on request to the HUNT Data Access Committee [hunt@medisin.ntnu.no]. The HUNT data access information describes the policy regarding data availability [https://www.ntnu.edu/hunt/data] .

## Abbreviations

AUC: area under the receiver operating characteristic curve
BMI: body mass index
BBDMM: blood-based DNA methylation marker model
CpG: Cytosine-phosphate-Guanine
EN: Elastic Net
ESTHER: *Epidemiologische Studie zu Chancen der Verhütung, Früherkennung und optimierten Therapie chronischer Erkrankungen in der älteren Bevölkerung*
EWAS: epigenome-wide association studies
HUNT: Trøndelag Health Study
LDCT: low-dose computed tomography
LC: lung cancer
OR: odds ratios
SD: standard deviation
