# Supplementary material for: Blood-based DNA methylation marker model for short-term and long-term lung cancer risk prediction
Source: BMC Med. 2026 Jun 6;24:344. doi: 10.1186/s12916-026-04973-y (PMC13242670; doi:10.1186/s12916-026-04973-y)
Supplement: Supplementary file 1 — Supplementary Figure 1: The cluster heatmap depicting β-methylation values from low (blue), intermediate (white) to high (red) in ESTHER participants by age, sex and outcome subgroups. [file 12916_2026_4973_MOESM1_ESM.docx]

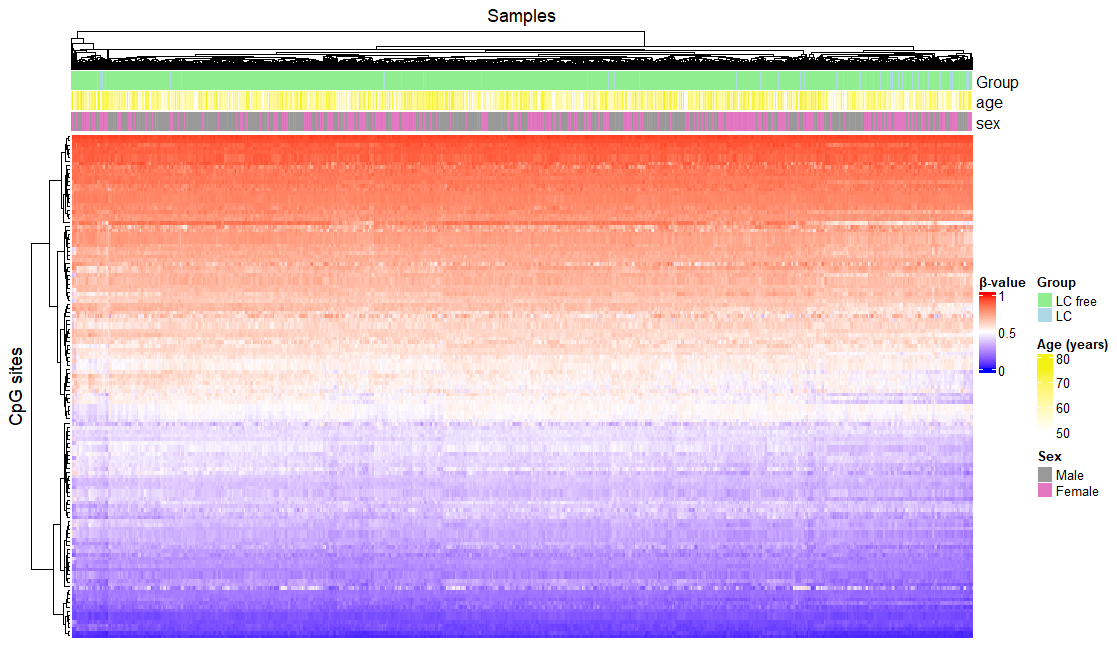


**Supplementary Figure 1:** The cluster heatmap depicting β-methylation values from low (blue), intermediate (white) to high (red) in ESTHER participants by age, sex and outcome subgroups. **Abbreviations**: **LC**- Lung cancer.
