# Supplementary material for: Blood-based DNA methylation marker model for short-term and long-term lung cancer risk prediction
Source: BMC Med. 2026 Jun 6;24:344. doi: 10.1186/s12916-026-04973-y (PMC13242670; doi:10.1186/s12916-026-04973-y)
Supplement: Supplementary file 2 — Supplementary Figure 2: Scatter-box-Violin plots showing distribution of the DNA methylation markers selected in the models among incident lung cancer cases and participants who did not develop LC from A. ESTHER, B. HUNT2 and C. HUNT3 population based cohorts. [file 12916_2026_4973_MOESM2_ESM.docx]

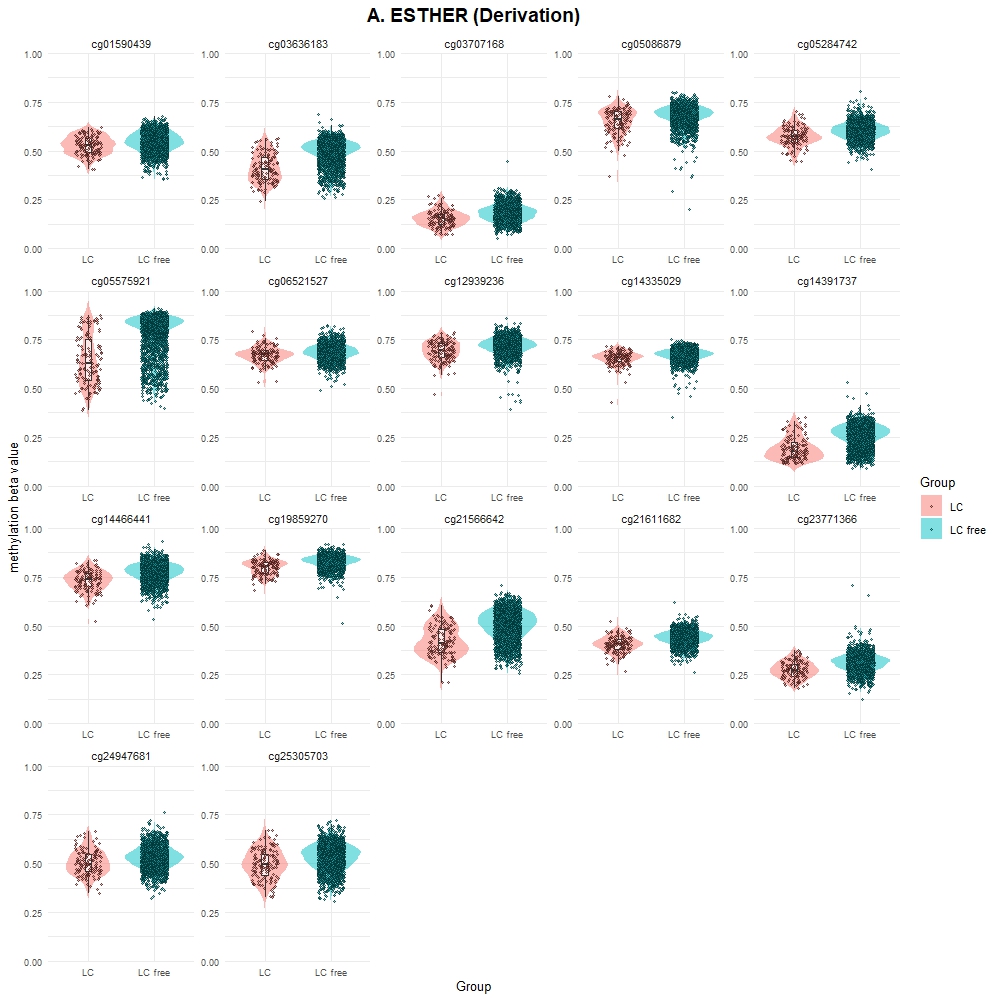


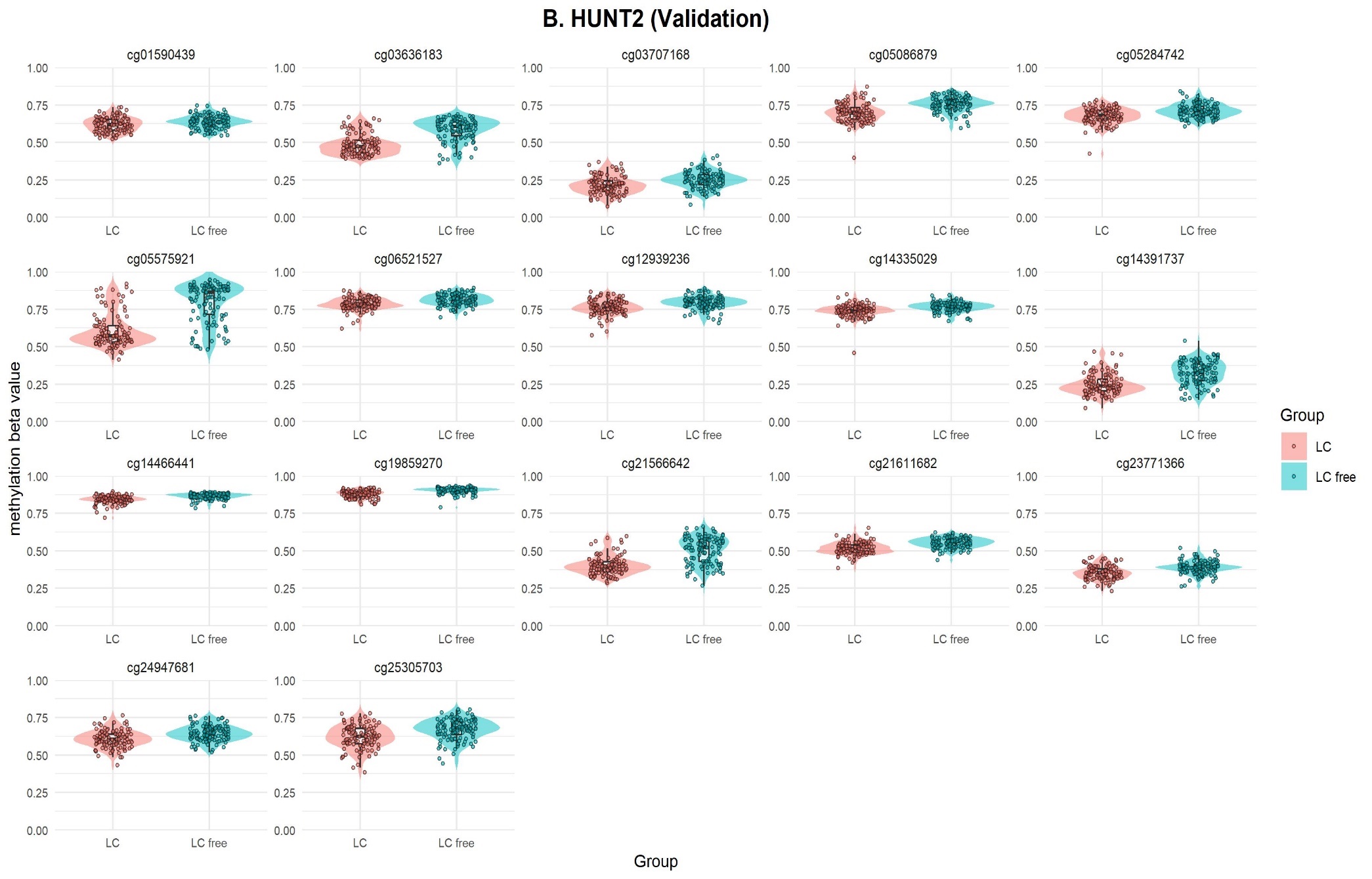


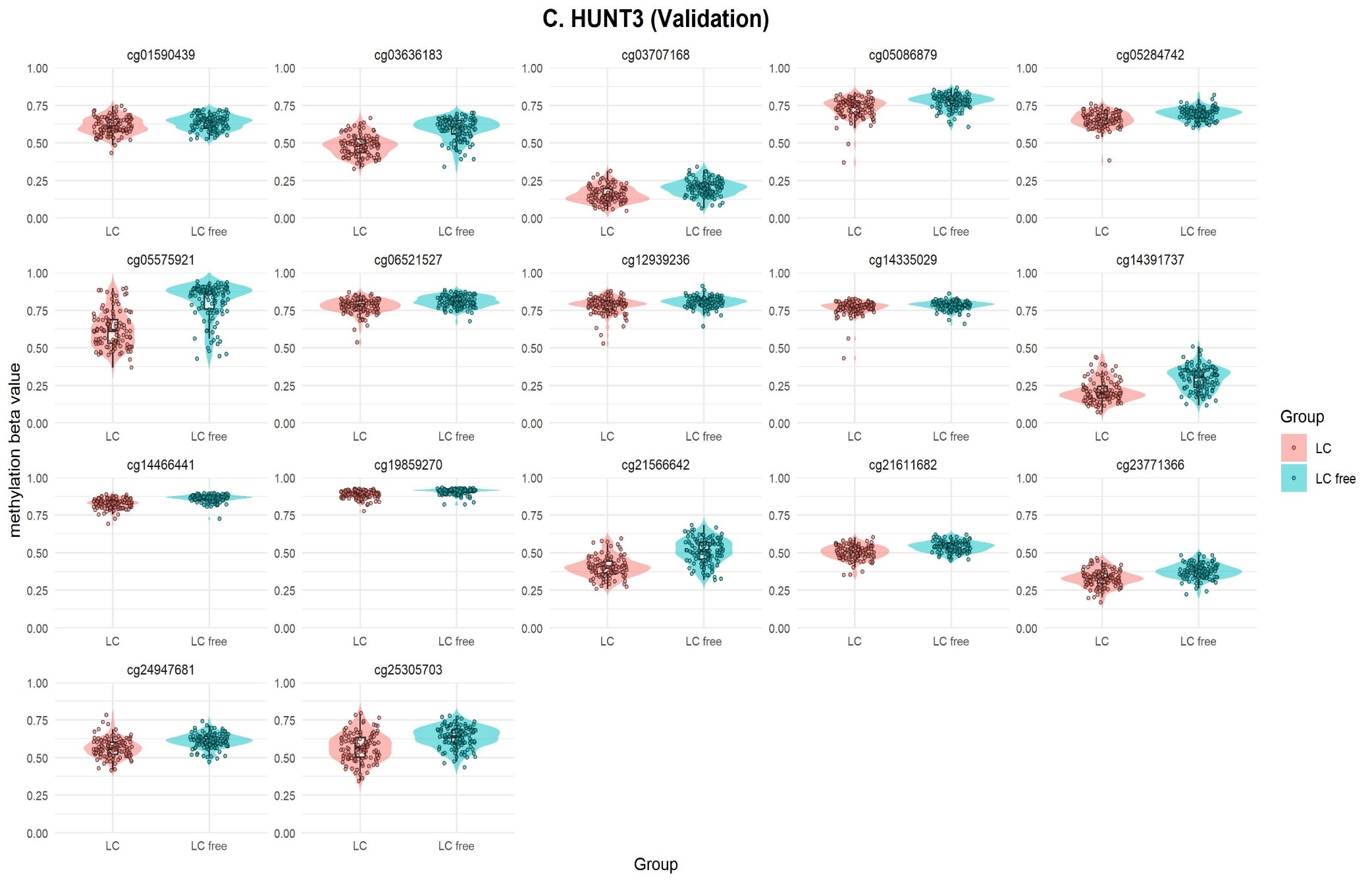


**Supplementary Figure 2:** Scatter-box-Violin plots showing distribution of the DNA methylation markers selected in the models among incident lung cancer cases and participants who did not develop LC from A. ESTHER, B. HUNT2 and C. HUNT3 population based cohorts. **Abbreviations**: **LC**- lung cancer.
