# Supplementary material for: Blood-based DNA methylation marker model for short-term and long-term lung cancer risk prediction
Source: BMC Med. 2026 Jun 6;24:344. doi: 10.1186/s12916-026-04973-y (PMC13242670; doi:10.1186/s12916-026-04973-y)
Supplement: Supplementary file 3 — Supplementary Figure 3: Area under the receiver operating characteristic curve of the blood-based DNA methylation marker model (BBDMM) among population subgroups by A. Age, B. Sex, C. BMI and D. Smoking status in the derivation and validation sets. [file 12916_2026_4973_MOESM3_ESM.docx]

1. **ROC curves by age**


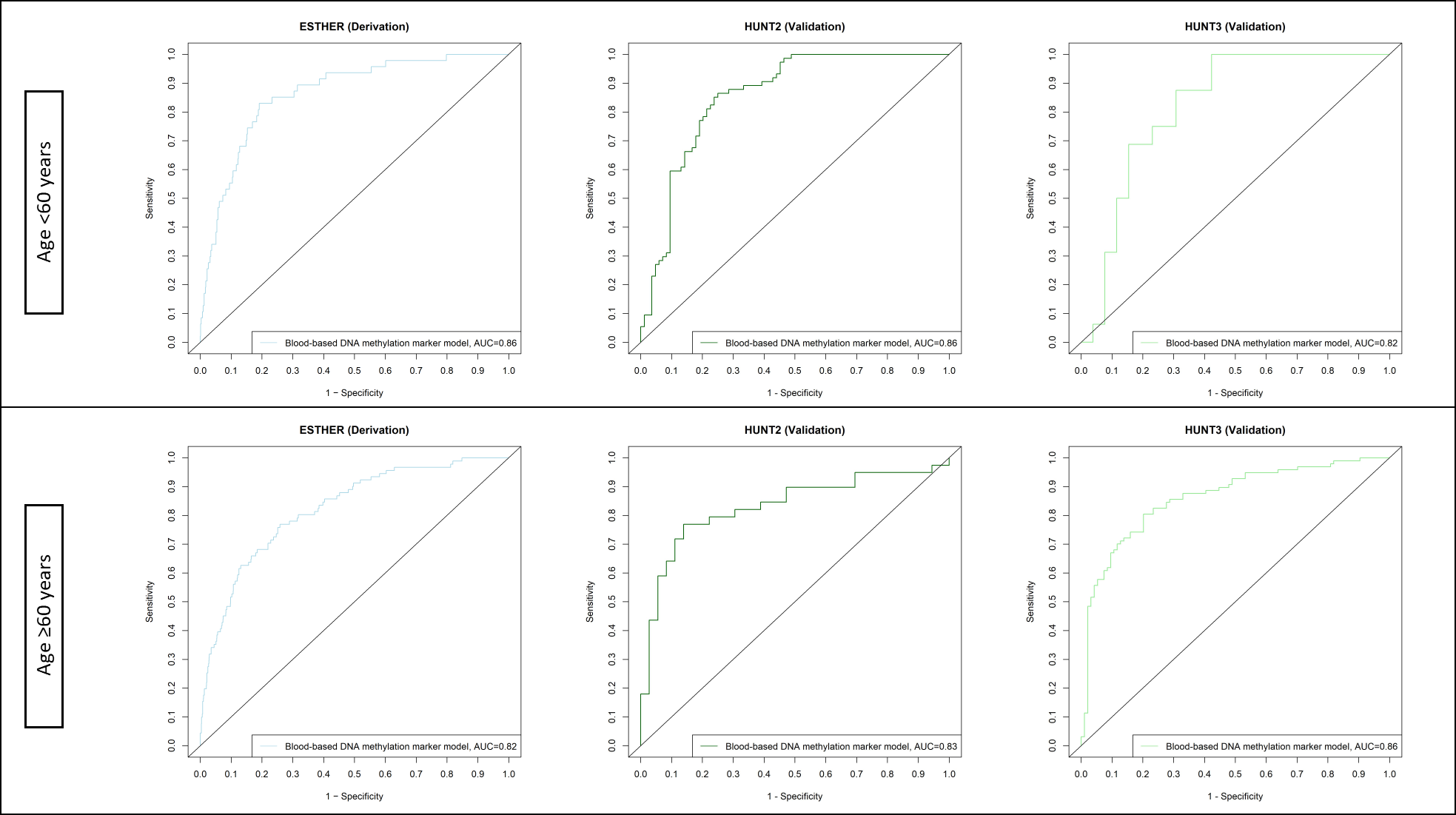


1. **ROC curves by sex**

**
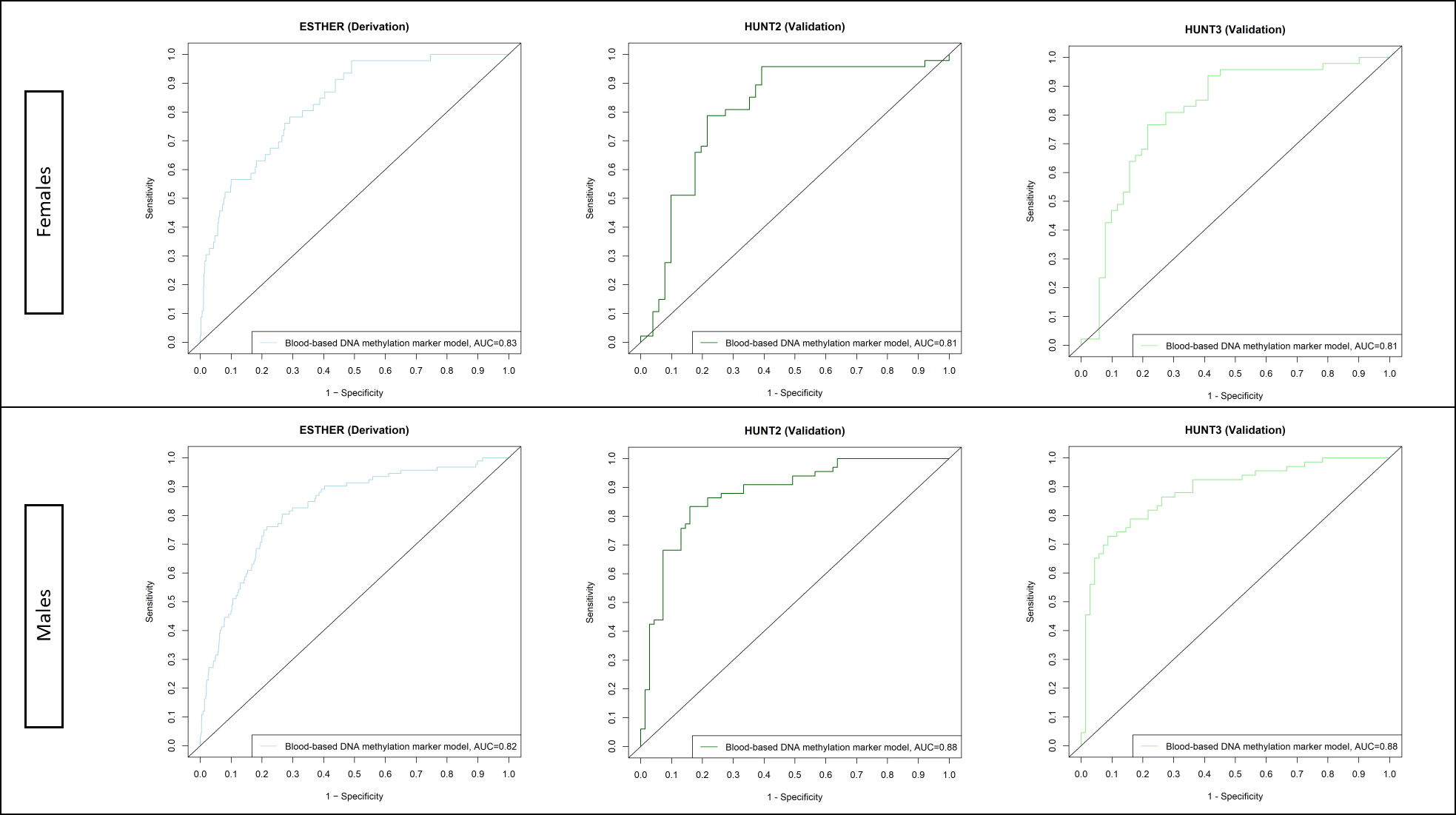
**

1. **ROC curves by BMI**

**
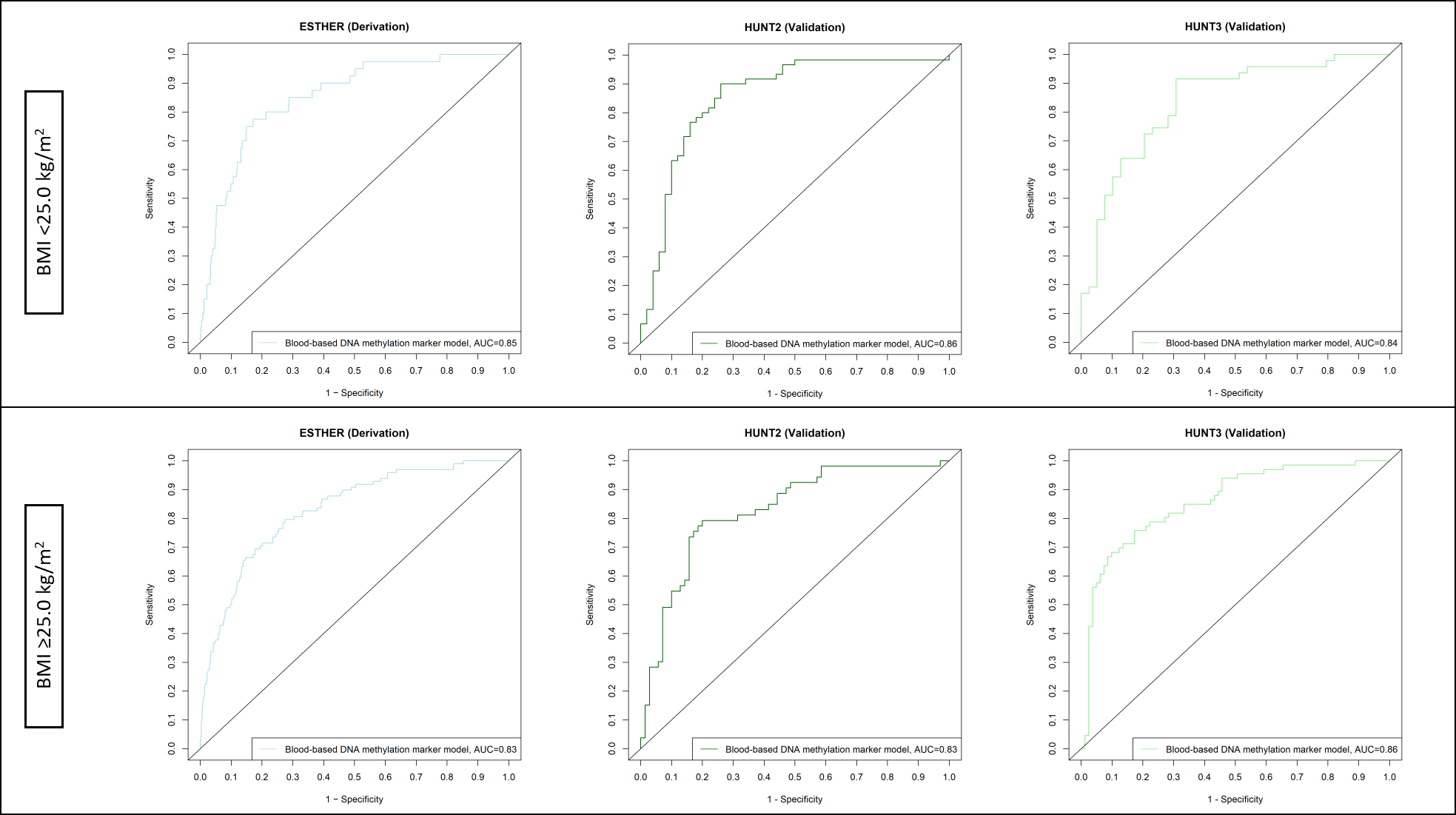
**

1. **ROC curves by smoking status**

**
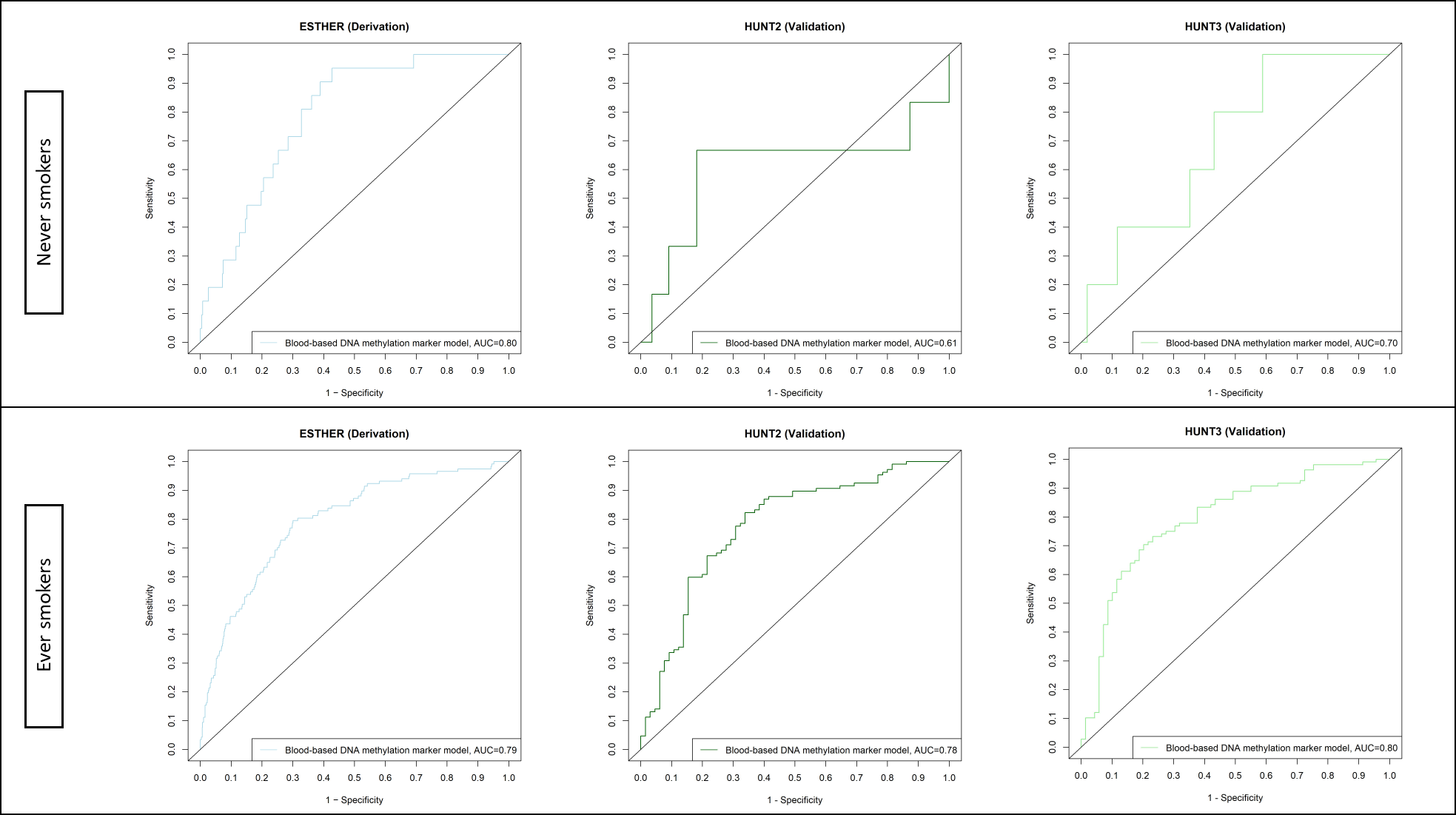
**

**Supplementary Figure 3:** Area under the receiver operating characteristic curve of the blood-based DNA methylation marker model (BBDMM) among population subgroups by A. Age, B. Sex, C. BMI and D. Smoking status in the derivation and validation sets.
