# Supplementary material for: Blood-based DNA methylation marker model for short-term and long-term lung cancer risk prediction
Source: BMC Med. 2026 Jun 6;24:344. doi: 10.1186/s12916-026-04973-y (PMC13242670; doi:10.1186/s12916-026-04973-y)
Supplement: Supplementary file 4 — Supplementary Table 1: EWAS identifying CpGs associated with LC risk [file 12916_2026_4973_MOESM4_ESM.docx]

**Supplementary Table 1:** EWAS identifying CpGs associated with LC risk

| **First author [ref]** | **Illumina Infinium Assay Type** | **CpG probes** | **Discovery set** | | | **Validation set** | | | **Number of CpGs identified associated with LC risk** |
| --- | --- | --- | --- | --- | --- | --- | --- | --- | --- |
|  |  |  | **Study** | **N LC** | **N Controls** | **Study** | **N LC** | **N Controls** |  |
| Fasanelli, 2015 [22] | HumanMethylation450 BeadChip | 450 K | NOWAC | 132 | 132 | MCCS  NSHDS  EPIC HD | 367  234  63 | 367  234  63 | 11 |
| Baglietto, 2017 [23] | HumanMethylation450 BeadChip | 450 K | EPIC Italy  MCCS | 185  367 | 185  367 | NOWAC  NSHDS  EPIC HD | 132  234  63 | 132  234  63 | 34 |
| Sandanger, 2018 [24] | HumanMethylation450 BeadChip | 450 K | NOWAC | 131 | 129 | NTR | 125 MZ pair  146 DZ pairs | | 25 |
| Battram*, 2019 [25] | HumanMethylation450 BeadChip | 450 K | EPIC Italy  MCCS  NOWAC  NSHDS | 185  367  132  324 | 185  367  132  324 | - | - | - | 16 |
| Sun, 2021 [26] | HumanMethylation EPIC BeadChip | 850 K | HUNT2 | 139 | 137 | HUNT3 | 131 | 135 | 50 |
| Zhao,2022 [27] | HumanMethylation EPIC BeadChip | 850 K | CLUE II | 208 | 222 | - | - | - | 16 |
| Petrovic, 2022 [28] | HumanMethylation450 BeadChip | 450 K | EPIC Italy  NOWAC | 185  128 | 512  314 | - | - | - | 29 |
| Faltus, 2022 [29] | HumanMethylation450 BeadChip | 450 K | EPIC HD | 66 | 66 | - | - | - | 12 |
| Domingo-Relloso, 2023 [30] | HumanMethylation EPIC BeadChip & HumanMethylation450 BeadChip | 850 K | SHS | 97 | 2224 | FHS | 56 | 2543 | 29 |

Total 146 unique CpGs and 123 available in ESTHER and HUNT

**CLUE II**- Campaign Against Cancer and Heart Disease; **DZ**- dizygotic; **EPIC HD**- European Prospective Investigation into Cancer and Nutrition Heidelberg lung cancer EWAS; **EWAS**- epigenome-wide association studies; **FHS**- Framingham Heart Study; **HUNT**- Trøndelag Health Study; **LC**- lung cancer; **MZ**- monozygotic; **MCCS**- Melbourne Collaborative Cohort Study; **NOWAC**- Norwegian Women and Cancer; **NSHDS**- the Northern Sweden Health and Disease Study; **SHS**- Strong Heart Study; **NTR**- Netherlands Twin Register.

*Meta-analysis of EWAS
