# Supplementary material for: Blood-based DNA methylation marker model for short-term and long-term lung cancer risk prediction
Source: BMC Med. 2026 Jun 6;24:344. doi: 10.1186/s12916-026-04973-y (PMC13242670; doi:10.1186/s12916-026-04973-y)
Supplement: Supplementary file 5 — Supplementary Table 2: Ability to predict incident lung cancer cases [file 12916_2026_4973_MOESM5_ESM.docx]

**Supplementary Table 2:** Ability to predict incident lung cancer cases

| **Predictors** | **ESTHER (Derivation)** | | **HUNT2 (Validation)** | | **HUNT3 (Validation)** | |
| --- | --- | --- | --- | --- | --- | --- |
|  | AUC (95%CI) | De-Long p-val | AUC (95%CI) | De-Long p-val | AUC (95%CI) | De-Long  p-val |
| BBDMM | 0.84 (0.80-0.87) | - | 0.85 (0.80-0.90) | - | 0.85 (0.80-0.90) | - |
| smoking status | 0.74 (0.70-0.78) | <0.0005 | 0.80 (0.74-0.85) | <0.05 | 0.77 (0.72-0.83) | <0.0005 |
| BBDMM+ smoking status | 0.84 (0.80-0.87) |  | 0.85 (0.80-0.90) |  | 0.86 (0.81-0.91) |  |

**Abbreviations**: **AUC**- area under the curve; **BBDMM-** blood-based DNA methylation marker model**; 95% CI**- 95 % confidence interval.
